# Orientation-Dependent Conversion of VLS-Grown Lead Iodide Nanowires into Organic-Inorganic Hybrid Perovskites

**DOI:** 10.3390/nano11010223

**Published:** 2021-01-16

**Authors:** Hyewon Shim, Yunjeong Hwang, Sung Gu Kang, Naechul Shin

**Affiliations:** 1 Department of Chemical Engineering, Inha University, Incheon 22212, Korea; simhw1396@inha.edu; 2 Program in Biomedical Science & Engineering, Inha University, Incheon 22212, Korea; ruflus16@inha.edu; 3 School of Chemical Engineering, University of Ulsan, Ulsan 44610, Korea; sgkang@ulsan.ac.kr

**Keywords:** PbI_2_, nanowires, vapor-liquid-solid, orientation, CH_3_NH_3_PbI_3_, perovskites

## Abstract

In this study, we demonstrate Sn-assisted vapor-liquid-solid (VLS) growth of lead iodide (PbI_2_) nanowires with van der Waals layered crystal structure and subsequent vapor-phase conversion into methylammonium lead iodide (CH_3_NH_3_PbI_3_) perovskites. Our systematic microscopic investigations confirmed that the VLS-grown PbI_2_ nanowires display two major growth orientations of [0001] and [$\bar{1}$2$\bar{1}$0], corresponding to the stacking configurations of PbI_2_ layers to the nanowire axis (transverse for [0001] vs. parallel for [$\bar{1}$2$\bar{1}$0]). The resulting difference in the sidewall morphologies was correlated with the perovskite conversion, where [0001] nanowires showed strong localized conversion at top and bottom, as opposed to [$\bar{1}$2$\bar{1}$0] nanowires with an evenly distributed degree of conversion. An ab initio energy calculation suggests that CH_3_NH_3_I preferentially diffuses and intercalates into ($11\bar{2}0$) sidewall facets parallel to the [$\bar{1}$2$\bar{1}$0] nanowire axis. Our results underscore the ability to control the crystal structures of van der Waals type PbI_2_ in nanowire via the VLS technique, which is critical for the subsequent conversion process into perovskite nanostructures and corresponding properties.

## 1. Introduction

The organic-inorganic hybrid perovskites (OIHPs) material system based on the metal halide structures (e.g., PbI_2_, PbBr_2_, SnI_2_, etc.) has been a focus of research interest, stemming from its superior physical properties such as high absorption coefficients and long carrier diffusion length [1,2]. The low cost of raw materials and the ease of preparation in which the crystallization is readily attainable in the solution phase at room temperature [3,4,5] have led the extensive studies regarding the OIHPs material to diverse applications including photonic [6,7,8], electronic [9,10], and optoelectronic [11,12] devices. Meanwhile, the efforts to fabricate OIHPs with various morphologies in low dimension forms also have been tried, especially in nanoparticles, thin films, and nanowires. Based on the general structure-property relationship, it is speculated that OIHPs in nanoscale would exhibit new optical/physical properties compared to their bulk counterpart, owing to the confinement effect [13,14]. One-dimensional (1D) nanowire is particularly interesting in modulating carrier transport and efficient charge separation with the controlling ability of radial and axial dimensions [15,16,17].

The vapor-liquid-solid (VLS) growth method has been widely used to fabricate one-dimensional (1D) nanostructures over the last several decades [18,19,20]. A variety of material systems, including group IV (Si, Ge) and III–V (InAs, GaP, etc.) semiconductors, have successfully demonstrated the VLS method as an effective approach to design and control nanowire structures [21,22]. Due to the characteristics of VLS mechanism employing metallic nanoparticles capable of forming eutectic alloys as growth catalysts, the growing materials take advantage of single crystallinity available at a lower temperature than their melting points, as well as the controllability over the growth directions, sidewall morphologies, compositions, etc. [23,24,25,26,27]. Recently, the application of VLS mechanism has been extended to the low-dimensional growth of other material systems, specifically layered semiconductors such as transition metal dichalcogenides (TMDs) [28,29], group IV chalcogenides [30,31], and metal halides [32,33]. Eda et al. reported the growth of two-dimensional (2D) molybdenum disulfide (MoS_2_) nanoribbons via the VLS method using molten Na-Mo-O droplet particles as the catalyst for crawling growth on the substrate [28]. Anisotropic van der Waals layered semiconductors, such as germanium (II) sulfide were also demonstrated to grow via VLS mechanism, as reported by Sutter et al. [30]. Our previous study showed that lead iodide (PbI_2_), another van der Waals type material system, can homoepitaxially grow on the PbI_2_ substrate layers pre-deposited on the muscovite mica surface [33]. These results emphasize the VLS mechanism as a universal approach to fabricate intrinsically two-dimensional van der Waals type crystals into one-dimensional nanowire structures, which may open up the new methodology to modulate their physical properties. For instance, the VLS-grown PbI_2_ nanowires can be converted into CH_3_NH_3_PbI_3_ (MAPbI_3_) perovskite nanowires via the introduction of CH_3_NH_3_I (MAI) in the vapor phase [32], of which the morphological characteristics are attractive in diverse photonic and optoelectronic applications including energy harvesting. However, there exists only a handful of information regarding the characteristics of VLS growth of lead halide nanowires and corresponding perovskite conversion process, which necessitates a systematic approach to understand the van der Waals layer growth in the radially confined nanowire domains and the effect of the stacking structure on the perovskite conversion process. Furthermore, studies on the diffusion and intercalation of vapor-phase MAI on the sidewall surfaces of 1D PbI_2_ nanostructures are yet to be reported.

In this regard, we demonstrate the VLS growth of PbI_2_ nanowires assisted by Sn nanoparticles and confirm that their axial orientations are classified into [0001] and [$\bar{1}$2$\bar{1}$0], which are closely related to the configuration of the 2H phase of van der Waals PbI_2_ layers in nanowire domains. Specifically, our systematic structural analysis indicated that the PbI_2_ layers stack differently for each growth direction (i.e., transverse for [0001] and parallel for [$\bar{1}$2$\bar{1}$0]), with discrete sidewall facet orientations. We correlated the sidewall configurations of each nanowire type with vapor-phase conversion characteristics into organic-inorganic hybrid perovskites (MAPbI_3_). It was found that the degree of perovskite conversion was locally different according to the nanowire growth direction, which is ascribed to the difference in the affinity of conversion precursor CH_3_NH_3_I (MAI) molecules to the exposed sidewall surface facets, as predicted by an ab initio energy calculation. These findings suggest a viable approach to controlling the nanostructures of lead halide-based perovskite materials for extensive use in various applications.

## 2. Materials and Methods

### 2.1. Sample Preparation and PbI_2_ Nanowire Growth

Both PbI_2_ nanowire growth and perovskite conversion were carried out in a home-built chemical vapor deposition (CVD) tube furnace system, as described elsewhere [33] and illustrated in the Supplementary Figure S1. First, Sn powders (Sigma Aldrich, St. Louis, Mo, USA, <150 nm in particle size, ≥99%) of 2.5 mg were dispersed in methanol (Duksan Reagents, Ansan, South Korea) in a vial and sonicated for 60 min to prepare a Sn suspension. A 2” diameter c-plane sapphire wafer (HI-Solar, Gwangju, South Korea, c-plane (0001), thickness 0.430 ± 0.025 mm, single-side polished) was cut to 5 mm × 18 mm, cleaned by rinsing with deionized water (DI), and dried with a nitrogen gun. Then, the substrate’s polished side was held down in contact with the Sn suspension surface for 1 min. The Sn-transferred substrate was immediately placed on a quartz boat (5 × 48 × 8 mm) with the contact side facing up, loaded into the CVD furnace, and positioned at 16 cm downstream from the center. PbI_2_ (Sigma Aldrich, St. Louis, Mo, USA, 99%) powder of 0.4 mg was loaded into another quartz boat and initially placed outside the heating zone. After vacuuming the system for 10 min at 1.6 Pa, Ar (Yuseong, Incheon, South Korea, 99.999%) carrier gas was introduced at the rate of 100 cm^3^/min and P = 2.8 × 10^4^ Pa. When the substrate temperature reached 300 °C, the precursor boat was moved into the heating zone, located 10 cm upstream from the furnace center. After 15 min, the precursor boat was removed from the heating zone to terminate the PbI_2_ nanowire growth.

### 2.2. Conversion to MAPbI_3_

The PbI_2_ nanowire sample was placed on a quartz boat for MAPbI_3_ conversion with the nanowire grown side facing up and placed 12 cm downstream from the furnace center. 50 mg of CH_3_NH_3_I (Sigma Aldrich, St. Louis, Mo, USA, 99%) powder was placed in another quartz boat and loaded into the system, initially outside the heating zone. The furnace was evacuated at 1.6 Pa for 10 min and set to 1.3 × 10^4^ Pa by flowing 100 cm^3^/min Ar. The temperatures of substrate and precursor were set to 130 °C and 150 °C, respectively. Once the substrate reached the desired temperature, the CH_3_NH_3_I boat was moved to the heating zone, and the conversion was continued for the desired times.

### 2.3. DFT Calculations

All density functional theory (DFT) calculations with dispersion corrections [34,35,36,37] were used to predict the adsorption strength of methylammonium iodide (CH_3_NH_3_I) on PbI_2_ surfaces of ($01\bar{1}0$) and ($11\bar{2}0$) using the Vienna Ab initio Simulation Package 5.4.1. (University of Vienna, Vienna, Austria) [38,39]. In this study, GGA-PBE [40] functionals were employed with a 400 eV cutoff. The force convergence criterion and the energy convergence criterion for all calculations were 0.01 eV/Å and 10^−4^ eV, respectively. 8 × 8 × 2 and 2 × 3 × 1 Monkhorst-Pack [41] k-point meshes were employed for bulk hexagonal PbI_2_ and its slabs (i.e., (1 × 3) surface unit cell for PbI_2_ ($01\bar{1}0$) and (1 × 2) surface unit cell for PbI_2_ ($11\bar{2}0$)), respectively. A 20 Å vacuum was included in PbI_2_ ($01\bar{1}0$) and PbI_2_ ($11\bar{2}0$) calculations. Out of 16 (7) layers in total, 9 (4) top layers were relaxed, and the rest of 7 (3) bottom layers were fixed for the PbI_2_ ($01\bar{1}0$) (PbI_2_ ($11\bar{2}0$)) surface. The dipole correction [42,43] in the z-direction was applied in this study. The hydrogen adsorption energy was obtained by [44]

(1) $$E_{\mathrm{ads}}^{{\mathrm{CH}}_{3}{\mathrm{NH}}_{3}I}=E_{{\mathrm{CH}}_{3}{\mathrm{NH}}_{3}I/{\mathrm{PbI}}_{2}}-E_{{\mathrm{CH}}_{3}{\mathrm{NH}}_{3}I}-E_{{\mathrm{PbI}}_{2}}$$

where $E_{\mathrm{ads}}^{{\mathrm{CH}}_{3}{\mathrm{NH}}_{3}I}$ refers to the adsorption energy of CH_3_NH_3_I on the PbI_2_ slabs. $E_{{\mathrm{CH}}_{3}{\mathrm{NH}}_{3}I/{\mathrm{PbI}}_{2}}$, $E_{{\mathrm{CH}}_{3}{\mathrm{NH}}_{3}I}$, and $E_{{\mathrm{PbI}}_{2}}$ represent the total energies of the adsorbed CH_3_NH_3_I on PbI_2_ slabs, CH_3_NH_3_I, and the PbI_2_ slabs, respectively. For geometry optimization of CH_3_NH_3_I, the cubic box (30 Å) was utilized.

### 2.4. Characterization

The morphologies of samples were measured using a field emission scanning electron microscope (Hitachi, Tokyo, Japan, SU 8010/S-4300), atomic force microscope with noncontact mode (Nanoscope Multimode Iva, Bruker, Billerica, MA, USA), and NX-10 AFM (Park system, Suwon, South Korea). PbI_2_ and MAPbI_3_ nanowires’ crystal structures were examined by a transmission electron microscope (JEOL, Akishima City, Japan, JEM2100F, 200 kV). A photoluminescence (EX-upright) spectrometer equipped with a charge-coupled device (CCD) optical microscope (Andor, Belfast, UK, DV420A-OE) was used to measure the optical properties of PbI_2_ and MAPbI_3_ with the laser wavelength of 532 nm with the power of 5 mW. For TEM and PL measurements, the nanowires were dry-transferred to a TEM grid (Ted Pella, Redding, CA, USA, #01810, Carbon Type-B, 200 mesh) or SiO_2_ substrate (300 nm thermal oxide layer on <100> Si, HI-solar, Gwangju, South Korea).

## 3. Results and Discussion

To accommodate anisotropic growth of PbI_2_ crystals, nanowire growth via the VLS method was employed in this study. It has been demonstrated that the self-catalyzed VLS mechanism using liquid Pb as a catalyst can be used for the growth of lead halides (PbX_2_, X = I, Br, or Cl) [32] as well as the inorganic perovskites (e.g., CsPbBr_3_) assisted by impurity alloying [45,46]. Sn nanoparticles are often used to facilitate the formation of catalyst droplets since Sn readily alloy with Pb to form a eutectic point at 183 ℃ with 39.1 wt% Pb. This low eutectic temperature enables VLS growth of PbI_2_ nanowires at the temperature conditions lower than the melting point (i.e., 327 °C) of pure Pb, which is generally desired to minimize the excessive PbI_2_ deposition on the vapor-solid interface during the overall nanowire growth process.

**Figure 1:**
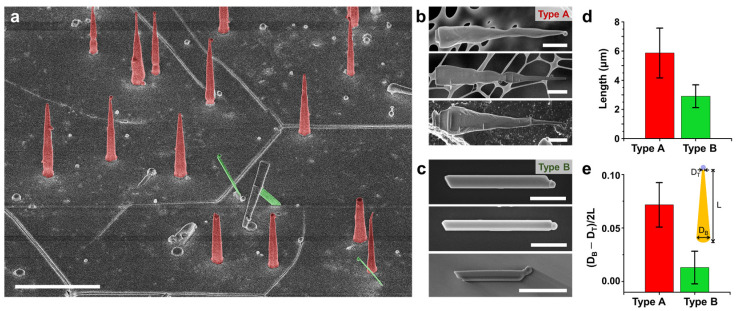
Sn-assisted, vapor-liquid-solid (VLS)-grown PbI_2_ nanowires on the [0001]-oriented c-sapphire substrate. (**a**) 45° view SEM image of PbI_2_ nanowires exhibiting two distinct morphologies: tapered, vertical nanowires (false-colored in red), and ribbon-like, tilted nanowires (false-colored in green). Scale bar, 5 μm. SEM image gallery of (**b**) vertical nanowires (denoted by type A) transferred onto a lacey carbon TEM grid, (**c**) kinked nanowires (denoted by type B) transferred to a Si wafer. Scale bars in (**b**,**c**), 1 μm. (**d**) Comparison of the nanowire lengths of type A and B, grown for 15 min. Average values are 5.87 μm and 2.90 μm for type A and B, respectively. (**e**) Difference between type A and B in the degree of tapering, defined as the difference in the radius of top and bottom divided by the nanowire length. D_B_ and D_T_ denote the bottom and top diameters, respectively.

Figure 1 demonstrates representative morphologies of PbI_2_ nanowires obtained by Sn-assisted VLS growth. The nanowires were grown on the [0001]-oriented c-sapphire substrate at T = 300 ℃ and P = 2.8 × 10^4^ Pa for 15 min under 100 cm^3^/min Ar carrier gas flow. First, Sn nanoparticles (d~150 nm on average) were transferred onto the sapphire substrate by dipping the substrate in the suspension for 30 s at room temperature, yielding the number density of 8.82/μm^2^, as shown in Supplementary Figure S2. Compared to the density of the nanowires (0.75/μm^2^) shown in Figure 1a, this value is significantly high and suggests that there exists a substantial energy barrier for the formation of catalytic Sn-Pb alloy droplet. It is noteworthy that no signature of 1D anisotropic growth was observed when PbI_2_ was deposited on the bare c-sapphire substrate without Sn particles (Supplementary Figure S3). This indicates that the 1D nanowire growth via precursor evaporation is promoted only when the Pb-Sn alloying occurs in the presence of Sn source (i.e., nanoparticles) unless excess Pb exists on the substrate by thermal evaporation or sputtering [32,33]. We also note that the as-grown PbI_2_ nanowires are primarily classified into two types with distinct morphology and orientation, as highlighted by red and green colors in Figure 1a. Vertically oriented nanowires with tapered sidewall morphologies are denoted by type A, while those grown tilted from the vertical direction showing straight sidewall morphologies are type B, as demonstrated in Figure 1b,c. Statistically, the length of type A nanowires (5.87 μm) are generally longer than that of the type B (2.90 μm). Considering that the anisotropic growth rate is highly contingent upon the interfacial surface energy of the growth front, our observation suggests that the crystallographic orientations of type A and B are dissimilar to each other, as discussed later. To quantitatively compare the difference in the sidewall morphology, we employed a degree of tapering, *σ*, which is defined as [47].

(2) $$\sigma =\frac{D_{B}-D_{T}}{2L}$$

where *D_B_* and *D_T_* refer to the diameter at the nanowire bottom and top, respectively, and *L* is the length. Figure 1e indicates that σ of type A (0.07) is significantly higher than that of type B (0.01), as reflected in the transmission electron microscopy (TEM) gallery showing nanowire sidewalls for both types (Supplementary Figure S4). We note that the tapered sidewalls of type A generally display an oscillatory decrease in diameter from bottom to top direction (Figure 1b and Supplementary Figure S4), as opposed to the straight sidewalls of type B, suggesting that the sidewalls of type A are faceted into multiple surface facets. Atomic force microscopy (AFM) analysis further supports the difference in the thickness profiles between each type, corroborating our SEM and TEM observations (Supplementary Figure S5). Again, the dissimilarity in sidewall morphology between type A and B strongly suggests that they exhibit distinct crystallographic characteristics.

**Figure 2:**
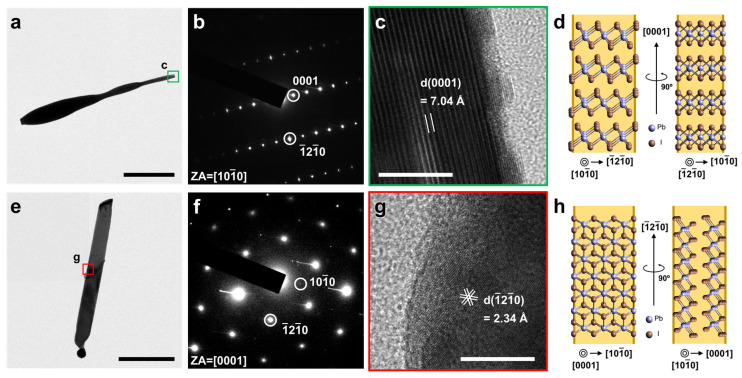
TEM investigations of the crystal structures for representative type A and type B PbI_2_ nanowires. Low-magnification bright-field TEM images of (**a**) type A and (**e**) type B nanowires. Scale bars, 1 μm. (**b**,**f**) Selected area electron diffraction pattern image corresponding to (**a**,**e**). (**c**,**g**) High-resolution TEM images of the nanowires denoted by the boxes in (**a**,**e**). Scale bars, 10 nm. Schematic illustrations of (**d**) type A and (**h**) type B PbI_2_ nanowire sidewall configurations.

We measured transmission electron microscopy (TEM) images of type A and B PbI_2_ nanowires to reveal their crystal structural difference. Figure 2a shows a representative type A nanowire with a tapered sidewall morphology, and the corresponding selected area electron diffraction (SAED) pattern in Figure 2b confirms that its axis is along [0001] orientation of 2H-phase PbI_2_, with the zone axis of [$10\bar{1}0$]. From Figure 2a, we note that the catalyst tip of this nanowire is absent, presumably due to the loss during the dry-transfer process of the as-grown nanowires onto the TEM grid. High-resolution TEM (HRTEM) image in Figure 2c confirms that the growth front of type A nanowire corresponds to the surface of the (0001) layer. The distance between each layer was measured as 7.04 Å, further supporting that the type A nanowire is comprised of the PbI_2_ layers stacked along with its [0001] axial orientation. Considering the three-fold symmetry of the hexagonal PbI_2_ crystal structure, we suspect that the sidewalls of type A nanowire are facing in axisymmetric ($11\bar{2}0$) or ($01\bar{1}0$) planes [48], as illustrated in Figure 2d. Low-magnification TEM images in Figure 2e confirm that the sidewall of a typical type B nanowire is untapered and straight, compared to that of type A. The growth direction was determined to be along [$\bar{1}$2$\bar{1}$0] orientation, as verified from the SAED image with the zone axis of [0001] (Figure 2f). In addition, the HRTEM image of the nanowire middle (Figure 2g) exhibits narrow lattice fringes (d~2.34 Å) relative to those observed from Figure 2c, which correspond to the interlayer spacing of ($11\bar{2}0$) plane of 2H PbI_2_ structure. Thus, we consider that the type B nanowire is composed of the (0001) PbI_2_ layer stacks parallel to the [$\bar{1}$2$\bar{1}$0] growth direction, which leads to the sidewalls of ($11\bar{2}0$) and (0001) planes (Figure 2h). We note that the difference in the sidewall morphologies between type A and B reflects the distinct surface energy of each sidewall configuration, which plays a crucial role in the vapor-phase conversion process to the organic-inorganic hybrid perovskite (OHP) structure (vide infra).

**Figure 3:**
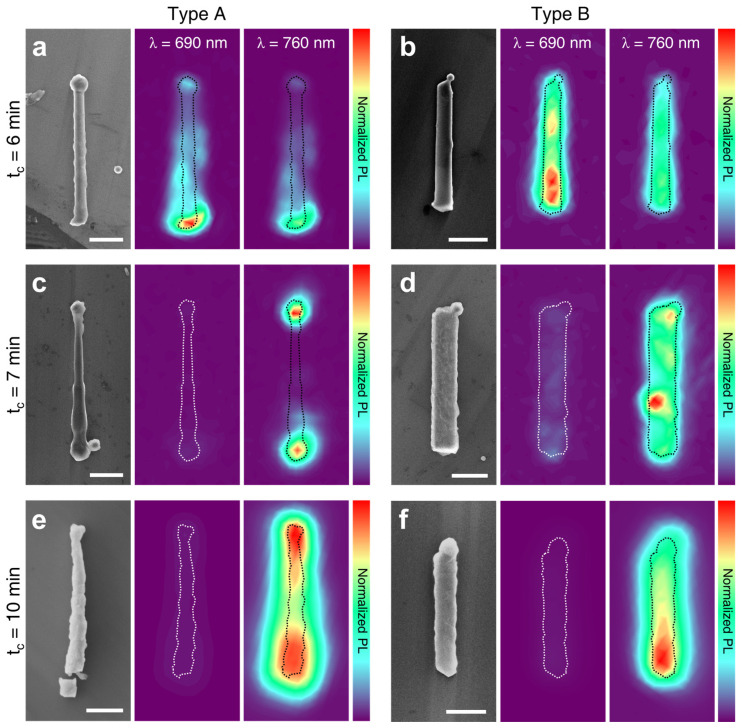
Time-dependent conversion from type A (left) and type B (right) PbI_2_ nanowires into CH_3_NH_3_PbI_3_ perovskites. Representative SEM images coupled with the photoluminescence (PL) intensity mappings at λ = 690 and 760 nm obtained from the conversion times of (**a**,**b**) 6 min, (**c**,**d**) 7 min, and (**e**,**f**) 10 min introducing vapor-phase CH_3_NH_3_I (MAI). All scale bars, 1 μm.

To understand the role of the nanowire axial orientations (and corresponding sidewall orientations) in the organic-inorganic hybrid perovskite conversion process, we exposed the substrate containing as-grown free standing PbI_2_ nanowires (both type A and B) to the vapor phase CH_3_NH_3_I (MAI). The nanowires were converted into CH_3_NH_3_PbI_3_ (MAPbI_3_) using 50 mg MAI at T = 130 ℃ and P = 1.3 × 10^4^ Pa under 100 cm^3^/min Ar flow, and the variations in sidewall morphology and corresponding optical properties as a function of the conversion time, t_c_, were monitored. Figure 3 demonstrates representative SEM images and photoluminescence (PL) intensity mappings at two different wavelengths (λ = 690 and 760 nm) of type A and B PbI_2_ nanowires exposed to MAI vapor for various t_c_ of 6, 7, and 10 min. It is notable that, as t_c_ increased, the nanowire sidewall surfaces became rougher for both type A (Figure 3a,c,e) and B (Figure 3b,d,f). Moreover, corresponding PL intensity mappings suggest that there exists a local difference in the degree of conversion, according to the nanowire axial orientation. When t_c_ = 6 min, the [0001]-oriented type A nanowire showed strong PL intensity at λ = 690 nm (intermediate between pure PbI_2_ at 518 nm and pure MAPbI_3_ at 760 nm) near the bottom, while the [$\bar{1}$2$\bar{1}$0]-oriented type B exhibited relatively even intensity distribution over the entire domain. Comparison of the intensity distribution at λ = 760 nm also supports that the conversion occurs more uniformly for type B than type A at the initial stage. As the conversion process continued to 7 min, the PL signal at 690 nm almost disappeared for type A, whereas the emission remained in the overall domain for type B, despite the weak intensity (Figure 3c,d). Importantly, the 760 nm emissions showed a clear local difference between type A and B. In type A, PL signals were observed only from the bottom and top region of the nanowire, while relatively even distribution was observed from type B, implying that MAI intercalation occurs in a different manner for type A and B, presumably due to the dissimilar surface diffusion behavior according to the sidewall facets. When the conversion time was further elongated to 10 min (Figure 3e,f), type A maintained intense 760 nm emission at the top and bottom region, as opposed to the type B with relatively regular distribution in the overall domain. At this stage, the 690 nm emissions became completely extinct for both types, indicating complete conversion into MAPbI_3_.

To correlate the local difference in the degree of conversion with the geometry between nanowires and substrate of each type, we compared the PL emission at the bottom of both type A and B according to the conversion times, as demonstrated in Supplementary Figure S6. Surprisingly, the trends of normalized PL shift at the nanowire bottom were analogous to each other, implying that the supply of MAI molecules to the free-standing PbI_2_ domains occurs similarly for both types via substrate surface diffusion. The strong localization of 760 nm emissions at the top of type A suggest that the perovskite conversion is also promoted by direct MAI impingement onto the catalyst droplet, which is unlikely for type B, where even distribution of PL intensity is observed. Thus, the difference in the degree of perovskite conversion between type A and B might be related to the accessibility of gaseous MAI onto the surface of nanowire sidewalls, necessitating the evaluation of MAI affinity to the exposed PbI_2_ surface facets since they must intercalate through the crystal layers. In our previous study, we reported that the [0001]-oriented PbI_2_ nanowires show three-fold symmetric sidewalls (i.e., truncated hexagons), which are primarily comprised of {$10\bar{1}0$}-type facets [33]. In accordance with this knowledge, we note that the type A nanowires observed in this work generally exhibited “tapered oscillatory” morphology, suggesting that the original {$10\bar{1}0$} sidewalls are multi-faceted into other planes [49]. Moreover, the nanowire is possibly rotated by multiples of 30° around the nanowire axis across the transverse twin boundaries, which is frequently observed from the van der Waals type of nanostructures [31], and thereby yielding the emergence of secondary surface such as ($11\bar{2}$0) plane. Conversely, the [$\bar{1}$2$\bar{1}$0]-oriented type B nanowires displayed ($11\bar{2}0$) and (0001) sidewalls, as previously reported [32]. Therefore, this discrepancy in the sidewall orientations is suspected to be responsible for different conversion aspect between the two types.

**Figure 4:**
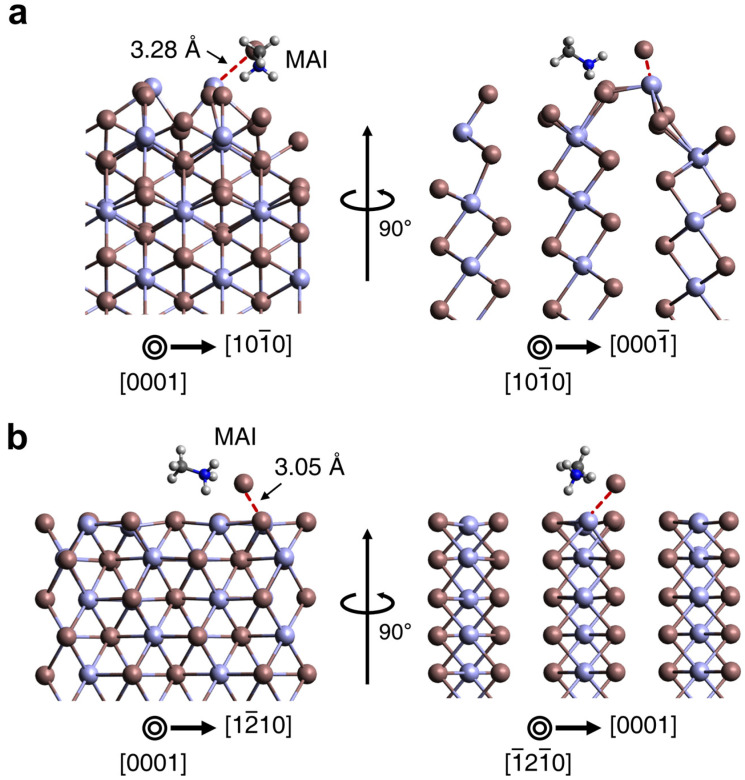
DFT-calculated atomic structures of MAI adsorption on the 2H PbI_2_ surface of (**a**) ($01\bar{1}0$) and (**b**) ($11\bar{2}0$) planes. Pb, I, C, N, and H atoms are illustrated using lavender, brown, blue, gray, and white colors, respectively.

The aforementioned observations led us to hypothesize that the degree of MAPbI_3_ conversion is dependent on the type of PbI_2_ sidewall facets where MAI inclusion occurs. The surface facets with high affinity for MAI may facilitate the conversion into MAPbI_3_, and their distribution in geometry would be crucial to determine the local degree of conversion. Thus, to confirm the difference of MAI inclusion to PbI_2_ lattice, we performed DFT calculations for the adsorption of MAI molecule on two different PbI_2_ planes of ($01\bar{1}$0) and ($11\bar{2}0$), as seen in Figure 4. We noted that the MAI adsorption onto the ($01\bar{1}0$) surface coincides with substantial surface reconstruction, yielding the adsorption distance of 3.28 Å (Figure 4a). We also noted that the diverse configurations are possible for MAI adsorption on ($01\bar{1}0$) surfaces (Supplementary Figure S7), although the adsorption presented in Figure 4a showed the most stable configuration. However, the I atom of MAI directly adsorbed on Pb atom exposed on the ($11\bar{2}0$) surface showing the adsorption distance of 3.05 Å without significant altering of the original lattice configuration, as illustrated in Figure 4b. Table 1 demonstrates the corresponding results of which the adsorption energy of MAI on PbI_2_ ($01\bar{1}0$) is more negative than that on PbI_2_ ($11\bar{2}0$), implying that MAI preferably adsorbs on PbI_2_ ($01\bar{1}0$) compared to ($11\bar{2}0$) surface. These results corroborate with our observation that type B shows locally more even perovskite conversion than type A, owing to the geometry of the ($11\bar{2}0$) sidewall planes continuously extending parallel to the nanowire axis. MAI reaching to the bottom of the free-standing PbI_2_ layers of type B can quickly diffuse along the nanowire axis and intercalate through any region of ($11\bar{2}0$) sidewalls. This is not the case for type A where the mixed facets of ($01\bar{1}0$) and ($11\bar{2}0$) lie transverse to the nanowire axis, which requires MAI to cross each PbI_2_ layer for subsequent conversion and eventually impede the axial conversion.

**Figure 5:**
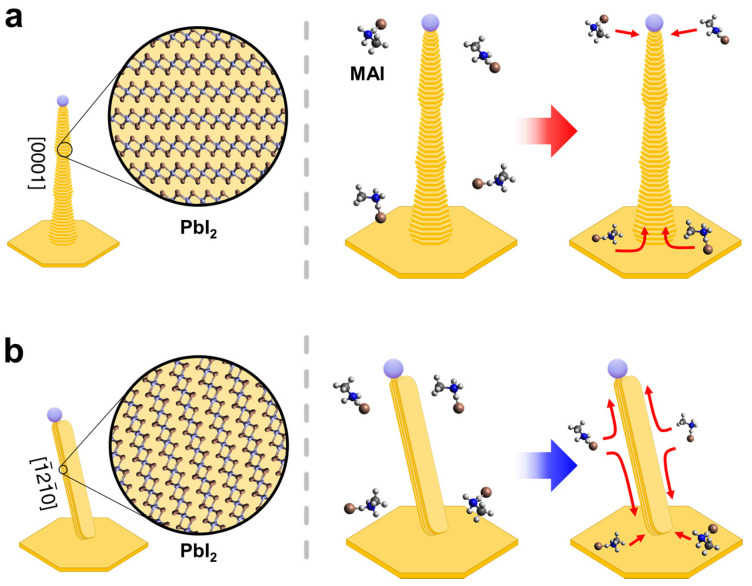
Schematics of vapor-phase perovskite conversion routes according to the growth direction of PbI_2_ nanowires. (**a**) [0001]-oriented, vertical type A nanowire composed of axial-transverse PbI_2_ layers. MAI sidewall diffusion is limited across the layers. (**b**) [$\bar{1}2\bar{1}0$]-oriented, tilted type B nanowire with parallel PbI_2_ layers along the axis. MAI diffuse along with the axial-parallel PbI_2_ layers and intercalate into the ($11\bar{2}0$) facets.

Based on our experimental observations and ab initio calculations, we propose a model of the vapor-phase conversion routes for type A and type B PbI_2_ nanowires. Figure 5 schematically illustrates the sidewall surface diffusion pathways of MAI according to the PbI_2_ nanowires growth direction. The supply of MAI to the type A nanowires primarily occurs by (1) direct absorption at the top and (2) surface diffusion from the substrate, as shown in Figure 5a. We noted that MAI sidewall diffusion along the nanowire axis was suppressed due to the transverse geometry of 2H PbI_2_ layers and eventually led to the localized perovskite conversion. However, type B nanowires with PbI_2_ layers parallel to the [$\bar{1}2\bar{1}0$] axial direction permits the MAI sidewall diffusion to occur for a larger distance (Figure 5b). MAI supply routes through both top absorption and bottom diffusion facilitate axial diffusion of MAI molecules and intercalation into ($11\bar{2}0$) sidewall facets. In this way, a uniform degree of perovskite conversion was observed for overall crystal domains of type B nanowires throughout the process.

It is worth mentioning that the continuous MAI supply eventually resulted in the complete conversion of both type A and B PbI_2_ nanowires into MAPbI_3_ structures. Supplementary Figure S8 demonstrates that both types display the signature of radial volume expansion after conversion time of 15 min, due to the complete MAI intercalation through sidewalls. TEM images measured on the representative samples confirm that the original single-crystalline PbI_2_ 2H phase was completely altered to the polycrystalline MAPbI_3_. SAED measurements conducted at two different regions of each nanowire further support that the vapor phase conversion ultimately leads to the formation of polycrystalline domains, regardless of the original nanowire orientation. Nevertheless, the systematic study of the nanowire orientation-dependent perovskites conversion provided here offers important insight to engineer the nanostructure and impart diverse functionalities to perovskite materials.

## 4. Conclusions

In conclusion, we have demonstrated that Sn-assisted VLS growth of PbI_2_ nanowires exhibits two different growth orientations of [0001] and [$\bar{1}2\bar{1}0$]. While [0001]-oriented, vertical nanowires generally show tapered and oscillatory sidewall morphologies, [$\bar{1}2\bar{1}0$]-oriented, tilt nanowires have straight sidewalls without significant tapering. Our extensive structural analysis confirms that their morphological difference is related to the geometry of [0001] 2H PbI_2_ layers relative to the axial orientation (i.e., parallel vs. transverse), which results in the local difference in the degree of hybrid perovskites (MAPbI_3_) conversion upon the exposure to vapor-phase MAI molecules. Combined with the ab initio energy calculation, we confirm that the vapor-phase conversion of PbI_2_ nanowires to MAPbI_3_ perovskites depends on the relative affinity of MAI to the sidewall facets and the arrangement of those facets in the domain. These results highlight the importance of leveraging metal halide nanowires for the organic-inorganic hybrid perovskites and provide insightful information to develop strategies exploiting perovskites nanostructures for a wide variety of applications.

## Figures and Tables

**Table 1:** Adsorption energy of CH_3_NH_3_I on PbI_2_ ($01\bar{1}0$) and PbI_2_ ($11\bar{2}0$) surfaces.

Surface	$E_{ads}^{CH_{3}NH_{3}I}\;(\mathrm{eV})$
PbI_2_ ($01\bar{1}0$)	−2.208
PbI_2_ ($11\bar{2}0$)	−1.525

## Data Availability

All data are available upon email request.

## Supplementary Materials

The following are available online at https://www.mdpi.com/2079-4991/11/1/223/s1, Figure S1: Schematic diagram of the CVD system used for PbI_2_ nanowire growth and MAPbI_3_ conversion, photographs of the substrate, and precursors boats. Pressure and temperature profiles. Figure S2: SEM image of Sn nanoparticles transferred onto c-plane sapphire after 30 s contact with Sn suspension with the number density statistics. Figure S3: SEM images and EDX mapping showing PbI_2_ platelet growth without the existence of Sn nanoparticles. Figure S4: TEM image gallery of the type A and type B PbI_2_ nanowires. Figure S5: AFM height profiles and images of the type A and type B PbI_2_ nanowires. Figure S6: Normalized PL spectra measured at the bottom regions of the type A and type B PbI_2_ nanowires according to the various MAI conversion times of 0, 5, 6, 7, and 10 min. Figure S7: Different configurations for MAI adsorption on PbI_2_ ($01\bar{1}0$) surfaces with corresponding adsorption energies. Figure S8: Representative tilt-view SEM images of MAPbI_3_ nanowires converted from the type A and type B PbI_2_ nanowires.

## References

[1] Xing G., Mathews N., Sun S., Lim S.S., Lam Y.M., Grätzel M., Mhaisalkar S., Sum T.C. (2013). Long-Range Balanced Electron- and Hole-Transport Lengths in Organic-Inorganic CH_3_NH_3_PbI_3_. Science.

[2] Dong Q., Fang Y., Shao Y., Mulligan P., Qiu J., Cao L., Huang J. (2015). Electron-Hole Diffusion Lengths > 175 μm in Solution-Grown CH_3_NH_3_PbI_3_ Single Crystals. Science.

[3] Wang G., Li D., Cheng H.C., Li Y., Chen C.Y., Yin A., Zhao Z., Lin Z., Wu H., He Q. (2015). Wafer-Scale Growth of Large Arrays of Perovskite Microplate Crystal for Functional Electronics and Optoelectronics. Sci. Adv..

[4] Lee W., Lee J., Yun H., Kim J., Park J., Choi C., Kim D.C., Seo H., Lee H., Yu J.W. (2017). High-Resolution Spin-on-Patterning of Perovskite Thin Films for a Multiplexed Image Sensor Array. Adv. Mater..

[5] Wu W., Wang X., Han X., Yang Z., Gao G., Zhang Y., Hu J., Tan Y., Pan A., Pan C. (2019). Flexible Photodetector Arrays Based on Patterned CH_3_NH_3_PbI_3−x_Cl_x_ Perovskite Film for Real-Time Photosensing and Imaging. Adv. Mater..

[6] Zhang N., Fan Y., Wang K., Gu Z., Wang Y., Ge L., Xiao S., Song Q. (2019). All-Optical Control of Lead Halide Perovskite Microlasers. Nat. Commun..

[7] Stylianakis M.M., Maksudov T., Panagiotopoulos A., Kakavelakis G., Petridis K. (2019). Inorganic and Hybrid Perovskite Based Laser Devices: A Review. Materials.

[8] Schlaus A.P., Spencer M.S., Zhu X. (2019). Light–Matter Interaction and Lasing in Lead Halide Perovskites. Acc. Chem. Res..

[9] Jana S., Carlos E., Panigrahi S., Martins R., Fortunato E. (2020). Toward Stable Solution-Processed High-Mobility p-Type Thin Film Transistors Based on Halide Perovskites. ACS Nano.

[10] She X.-J., Chen C., Divitini G., Zhao B., Li Y., Wang J., Orri J.F., Cui L., Xu W., Peng J. (2020). A solvent-based surface cleaning and passivation technique for suppressing ionic defects in high-mobility perovskite field-effect transistors. Nat. Electron..

[11] Jaramillo-Quintero O.A., Sanchez R.S., Rincon M., Mora-Sero I. (2015). Bright Visible-Infrared Light Emitting Diodes Based on Hybrid Halide Perovskite with Spiro-OMeTAD as a Hole-Injecting Layer. J. Phys. Chem. Lett..

[12] Han H., Jeong B., Park T.H., Cha W., Cho S.M., Kim Y., Kim H.H., Kim D., Ryu D.Y., Choi W.K. (2019). Highly Photoluminescent and Environmentally Stable Perovskite Nanocrystals Templated in Thin Self Assembled Block Copolymer Films. Adv. Funct. Mater..

[13] Sapori D., Kepenekian M., Pedesseau L., Katan C., Even J. (2016). Quantum Confinement and Dielectric Profiles of Colloidal Nanoplatelets of Halide Inorganic and Hybrid Organic−Inorganic Perovskites. Nanoscale.

[14] Zhang D., Gu L., Zhang Q., Lin Y., Lien D.H., Kam M., Poddar S., Garnett E.C., Javey A., Fan Z. (2019). Increasing Photoluminescence Quantum Yield by Nanophotonic Design of Quantum-Confined Halide Perovskite Nanowire Arrays. Nano Lett..

[15] Jiang X., Xiong Q., Nam S., Qian F., Li Y., Lieber C.M. (2007). InAs/InP radial nanowire heterostructures as high electron mobility devices. Nano Lett..

[16] Kempa T.J., Tian B., Kim D.R., Hu J., Zheng X., Lieber C.M. (2008). Single and tandem axial pin nanowire photovoltaic devices. Nano Lett..

[17] Um H.D., Moiz S.A., Park K.T., Jung J.Y., Jee S.W., Ahn C.H., Kim D.C., Cho H.K., Kim D.W., Lee J.H. (2011). Highly selective spectral response with enhanced responsivity of n-ZnO/p-Si radial heterojunction nanowire photodiodes. Appl. Phys. Lett..

[18] Wagner R.S., Ellis W.C. (1964). Vapor-Liquid-Solid Mechanism of Single Crystal Growth. Appl. Phys. Lett..

[19] Morales A.M., Lieber C.M. (1998). A Laser Ablation Method for the Synthesis of Crystalline Semiconductor Nanowires. Science.

[20] Shin N., Chi M., Filler  M.A. (2014). Interplay between Defect Propagation and Surface Hydrogen in Silicon Nanowire Kinking Superstructures. ACS Nano.

[21] Schmidt V., Wittemann J.V., Gösele U. (2010). Growth, Thermodynamics, and Electrical Properties of Silicon Nanowires. Chem. Rev..

[22] Barrigón E., Heurlin M., Bi Z., Monemar B., Samuelson L. (2019). Synthesis and Applications of III-V Nanowires. Chem. Rev..

[23] Madras P., Dailey E., Drucker J. (2009). Kinetically Induced Kinking of Vapor−Liquid−Solid Grown Epitaxial Si Nanowires. Nano Lett..

[24] Sun Z., Siedman D.N., Lauhon L.J. (2017). Nanowire Kinking Modulates Doping Profiles by Reshaping the Liquid–Solid Growth Interface. Nano Lett..

[25] Kim S., Hill D.J., Pinion C.W., Christesen J.D., McBride J.R., Cahoon J.F. (2017). Designing Morphology in Epitaxial Silicon Nanowires: The Role of Gold, Surface Chemistry, and Phosphorus Doping. ACS Nano.

[26] Güniat L., Martí-Sánchez S., Garcia O., Boscardin M., Vindice D., Tappy N., Friedl M., Kim W., Zamani M., Francaviglia L. (2019). III–V Integration on Si(100): Vertical Nanospades. ACS Nano.

[27] Johansson J., Dick K.A. (2011). Recent advances in semiconductor nanowire heterostructures. CrystEngComm.

[28] Li S., Lin Y.-C., Zhao W., Wu J., Wang Z., Hu Z., Shen Y., Tang D.-M., Wang J., Zhang Q. (2018). Vapor-Liquid-Solid Growth of Monolayer MoS_2_ Nanoribbons. Nat. Mater..

[29] Huang L., Thi Q.H., Zheng F., Chen X., Chu Y.W., Lee C.-S., Zhao J., Ly T.H. (2020). Catalyzed Kinetic Growth in Two-Dimensional MoS_2_. J. Am. Chem. Soc..

[30] Sutter P., Wimer S., Sutter E. (2019). Chiral twisted van der Waals nanowires. Nature.

[31] Sutter E., French J.S., Sutter S., Idrobo J.C., Sutter P. (2020). Vapor–liquid–solid Growth and Optoelectronics of Gallium Sulfide van der Waals Nanowires. ACS Nano.

[32] Meyers J.K., Kim S., Hill D.J., Cating E.E., Williams L.J., Kumbhar A.S., McBride J.R., Papanikolas J.M., Cahoon J.F. (2017). Self-Catalyzed Vapor−Liquid−Solid Growth of Lead Halide Nanowires and Conversion to Hybrid Perovskites. Nano Lett..

[33] Shim H., Shin N. (2019). VLS Homoepitaxy of Lead Iodide Nanowires for Hybrid Perovskite Conversion. J. Phys. Chem. Lett..

[34] Grimme S., Antony J., Ehrlich S., Krieg H. (2010). A consistent and accurate ab initio parametrization of density functional dispersion correction (DFT-D) for the 94 elements H-Pu. J. Chem. Phys..

[35] Grimme S., Ehrlich S., Goerigk L. (2011). Effect of the damping function in dispersion corrected density functional theory. J. Comput. Chem..

[36] Becke A.D., Johnson E.R. (2005). A density-functional model of the dispersion interaction. J. Chem. Phys..

[37] Johnson E.R., Becke A.D. (2006). A post-Hartree-Fock model of intermolecular interactions: Inclusion of higher-order corrections. J. Chem. Phys..

[38] Kresse G., Furthmüller J. (1996). Efficient Iterative Schemes for Ab Initio Total-Energy Calculations Using a Plane-wave Basis Set. Phys. Rev. B.

[39] Kresse G., Furthmüller J. (1996). Efficiency of Ab-initio Total Energy Calculations for Metals and Semiconductors Using a Plane-wave Basis Set. Comput. Mater. Sci..

[40] Perdew J.P., Burke K., Ernzerhof M. (1996). Generalized Gradient Approximation Made Simple. Phys. Rev. Lett..

[41] Monkhorst H.J., Pack J.D. (1976). Special Points for Brillouin-Zone Integrations. Phys. Rev. B.

[42] Bengtsson L. (1999). Dipole Correction for Surface Supercell Calculations. Phys. Rev. B Condens. Matter Mater. Phys..

[43] Neugebauer J., Scheffler M. (1992). Adsorbate-Substrate and Adsorbate-Adsorbate Interactions of Na and K Adlayers on Al(111). Phys. Rev. B Condens. Matter Mater. Phys..

[44] Sholl D.S., Steckel J.A. (2009). Density Functional Theory: A Practical Introduction.

[45] Meng Y., Lan C., Li F., Yip S., Wei R., Kang X., Bu X., Dong R., Zhang H., Ho J.C. (2019). Direct Vapor-Liquid-Solid Synthesis of All-Inorganic Perovskite Nanowires for High-Performance Electronics and Optoelectronics. ACS Nano.

[46] Meng Y., Lai Z., Li F., Wang W., Yip S., Quan Q., Bu X., Wang F., Bao Y., Hosomi T. (2020). Perovskite Core–Shell Nanowire Transistors: Interfacial Transfer Doping and Surface Passivation. ACS Nano.

[47] Wang Y., Schmidt V., Senz S., Gösele U. (2006). Epitaxial growth of silicon nanowires using an aluminum catalyst. Nat. Nanotechnol..

[48] Zhong M., Zhang S., Huang L., You J., Wei Z., Liu X., Li J. (2017). Large-Scale 2D PbI_2_ Monolayers: Experimental Realization and Their Indirect Band-Gap Related Properties. Nanoscale.

[49] Ross F.M., Tersoff J., Reuter M.C. (2005). Sawtooth Faceting in Silicon Nanowires. Phys. Rev. Lett..

