# Supplementary material for: Orientation-Dependent Conversion of VLS-Grown Lead Iodide Nanowires into Organic-Inorganic Hybrid Perovskites
Source: Nanomaterials (Basel). 2021 Jan 16;11(1):223. doi: 10.3390/nano11010223 (PMC7830942; doi:10.3390/nano11010223)
Supplement: Supplementary file 1 [file nanomaterials-11-00223-s001.pdf]

SUPPELMENTARY INFORMATION

Orientation-Dependent Conversion of VLS-Grown Lead Iodide Nanowires into Organic-Inorganic Hybrid Perovskites

Hyewon Shim [1], Yunjeong Hwang [2], Sung Gu Kang [3] and Naechul Shin [1,2,*]

1 Department of Chemical Engineering, Inha University, Incheon 22212, Korea; simhw1396@inha.edu
2 Program in Biomedical Science & Engineering, Inha University, Incheon 22212, Korea; ruflus16@inha.edu
3 School of Chemical Engineering, University of Ulsan, Ulsan 44610, Korea; sgkang@ulsan.ac.kr
* Correspondence: nshin@inha.ac.kr

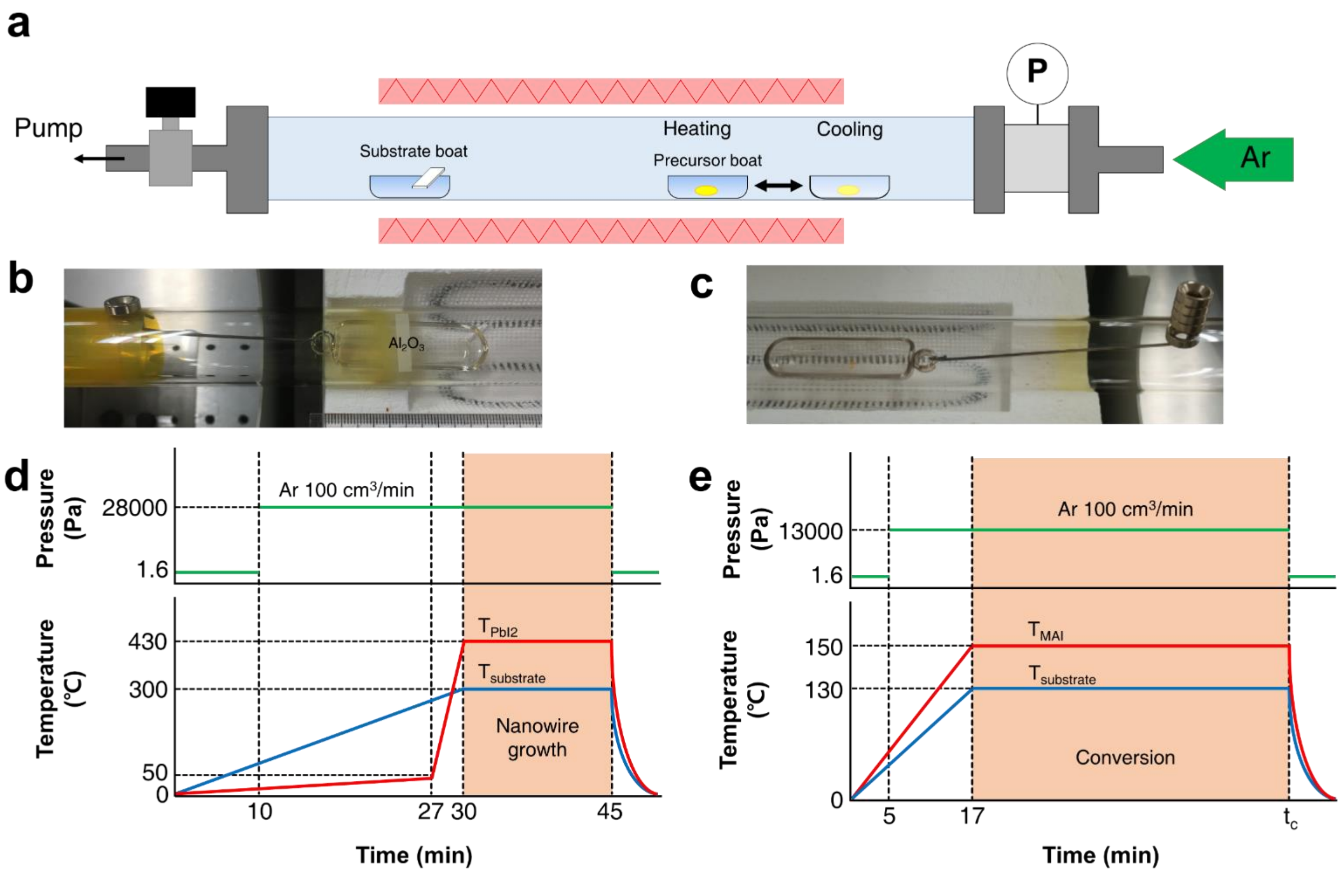

Figure S1. **(a)** Schematic diagram of the CVD system used for PbI_2 nanowire growth and perovskite conversion. Photograph images of **(b)** the substrate boat and **(c)** the precursor boat. Pressure and temperature profiles as a function of time corresponding to **(d)** PbI_2 nanowire growth, **(e)** perovskite conversion to $MAPbI_3$.

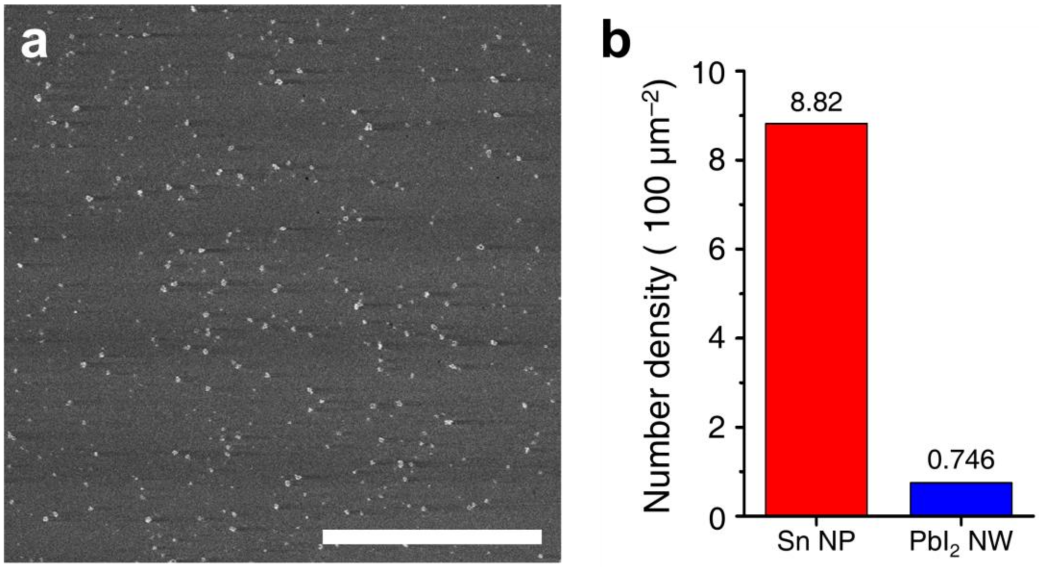

Figure S2. **(a)** SEM image of Sn nanoparticles transferred onto c-plane sapphire after 30 s contact with Sn suspension. Scale bar, 30 μm. (**b**) Statistics of the number density per 100 μm^2 of the Sn nanoparticles and the as-grown PbI_2 nanowires.

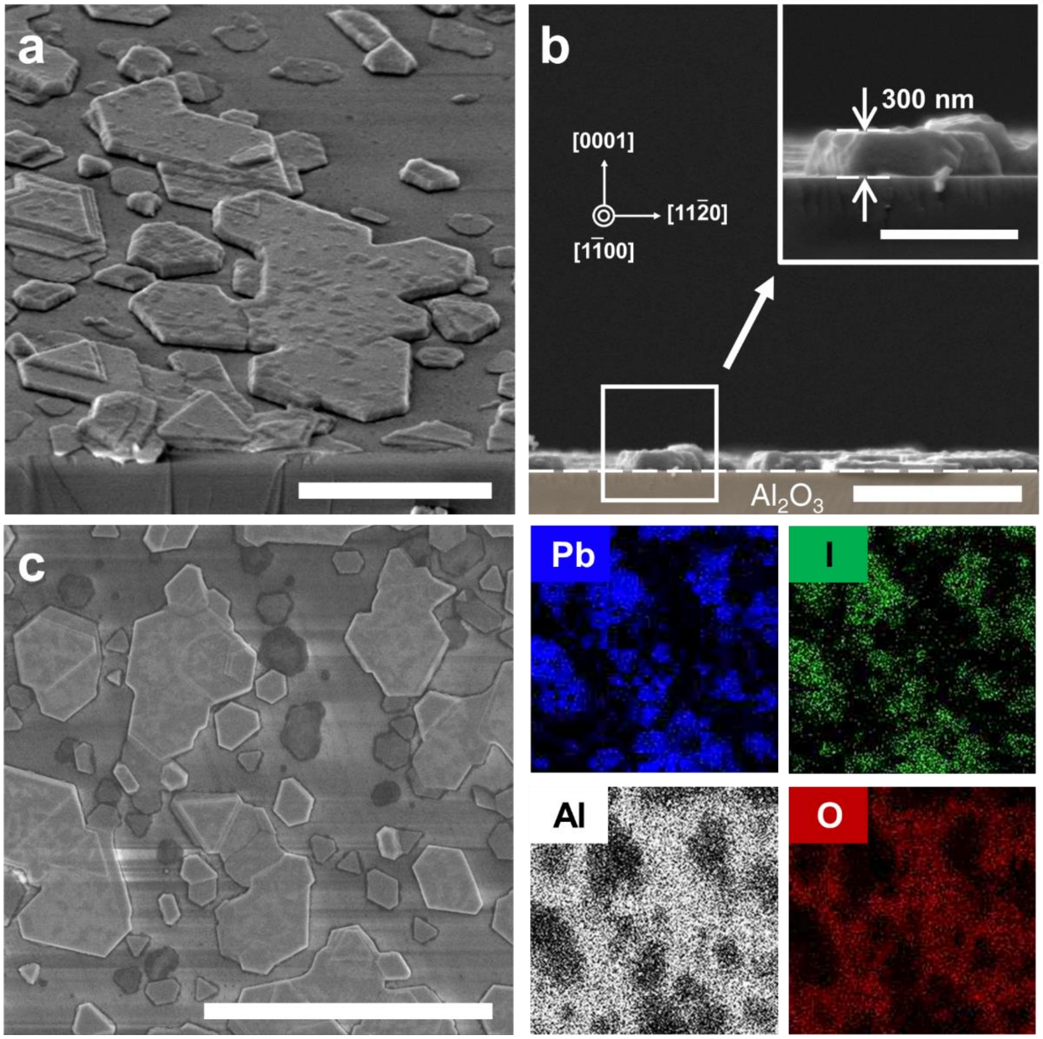

Figure S3. PbI_2 crystals grown without Sn nanoparticle. **(a)** Tilt-view SEM image of the PbI_2 platelets, obtained from the direct growth of PbI_2 on c-sapphire (0001) substrate without Sn nanoparticles. Scale bar, 5 μm. **(b)** Side-view SEM image of the sample in (a), exhibiting island growth. Scale bar, 2 μm. Inset in (b) is a magnified SEM image showing the average height of the PbI_2 island is about 300 nm. Scale bar, 1 μm. **(c)** Top-view SEM image (left) and corresponding EDS mapping images (right) of PbI_2 crystals on c-sapphire (0001) substrate. Hexagonal or truncated triangular PbI_2 platelet domains are clearly visible on the substrate. Scale bar, 10 μm.

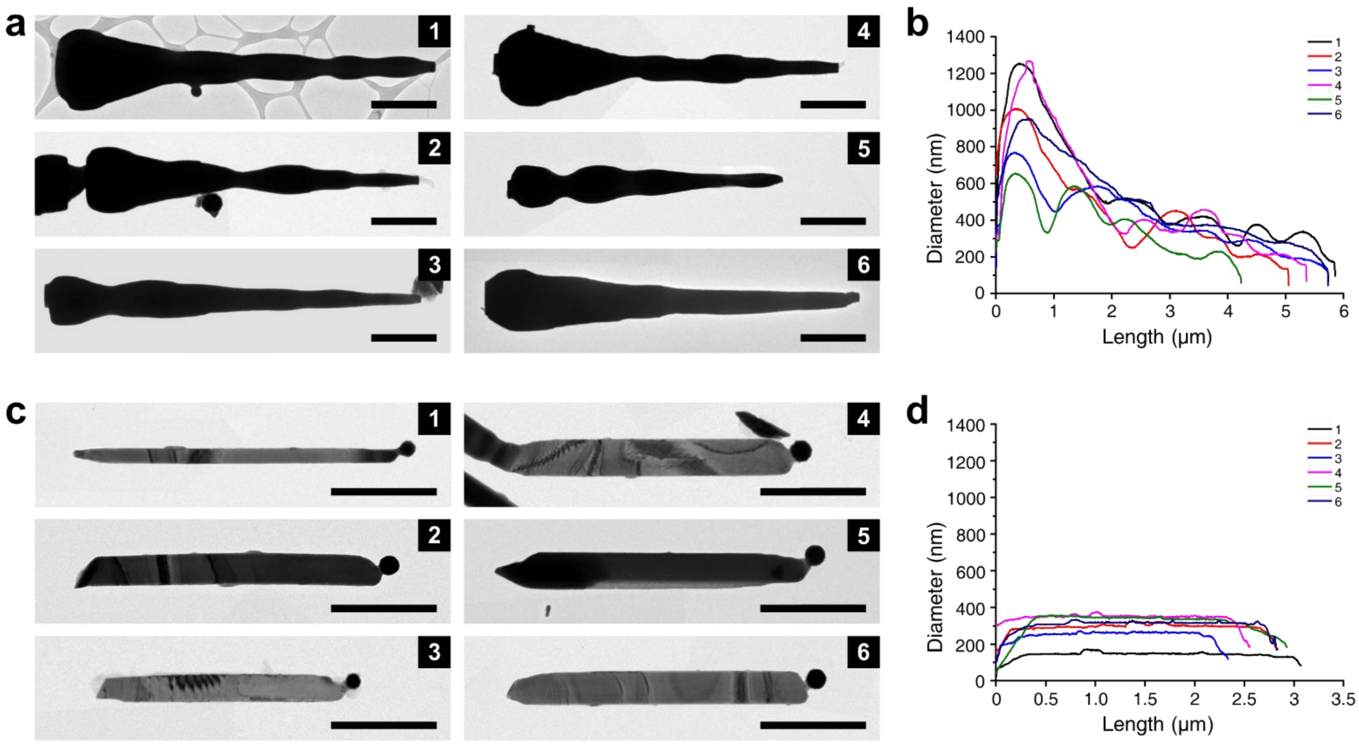

Figure S4. (a) TEM image gallery of the type A nanowires. Scale bars, 1 μm. (b) Corresponding diameter profile extracted from TEM images demonstrating tapered oscillatory sidewall morphologies. (c) TEM images of the type B nanowires. Scale bars, 1 μm. (d) Corresponding diameter profile extracted from TEM images showing straight sidewall morphologies.

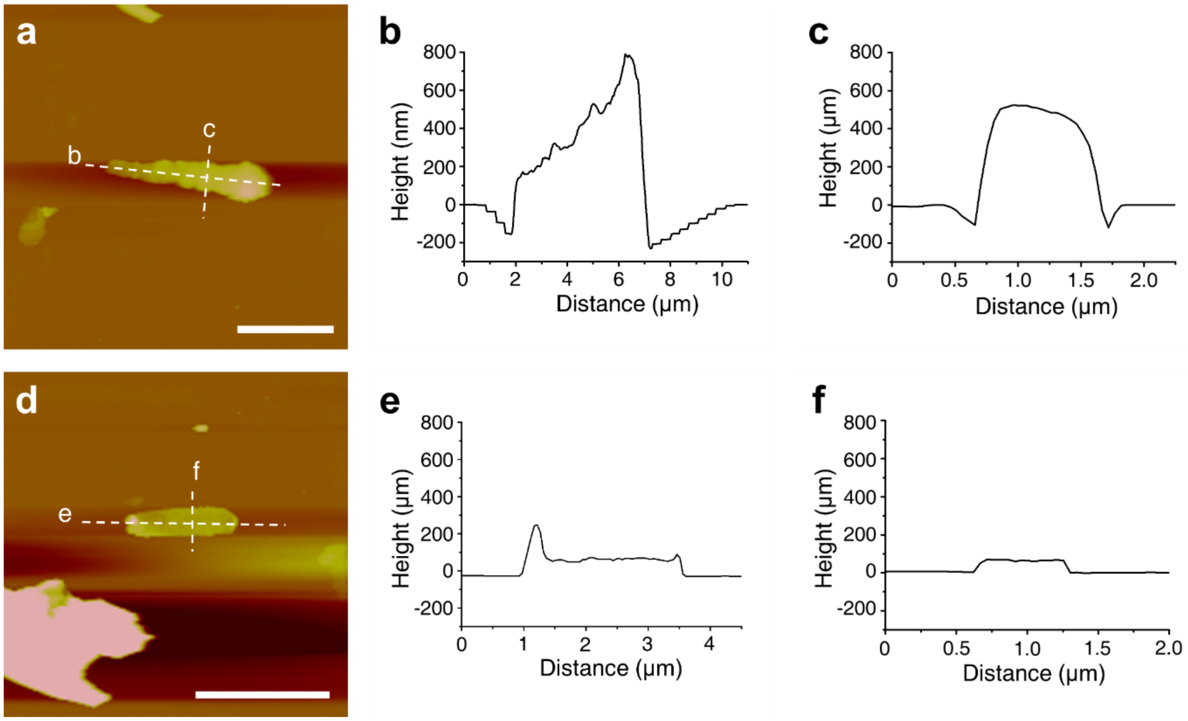

Figure S5. (a) AFM height image of a representative type A nanowire. Scale bar, 3 μm. (b) Longitudinal line profile indicated by a dotted line in (a). (c) Transverse line profile indicated by dotted line in (a). (d) AFM height image of a representative type B nanowire. Scale bar, 3 μm. (e) Longitudinal line profile indicated by dotted line in (d). (f) Transverse line profile indicated by dotted line in (d).

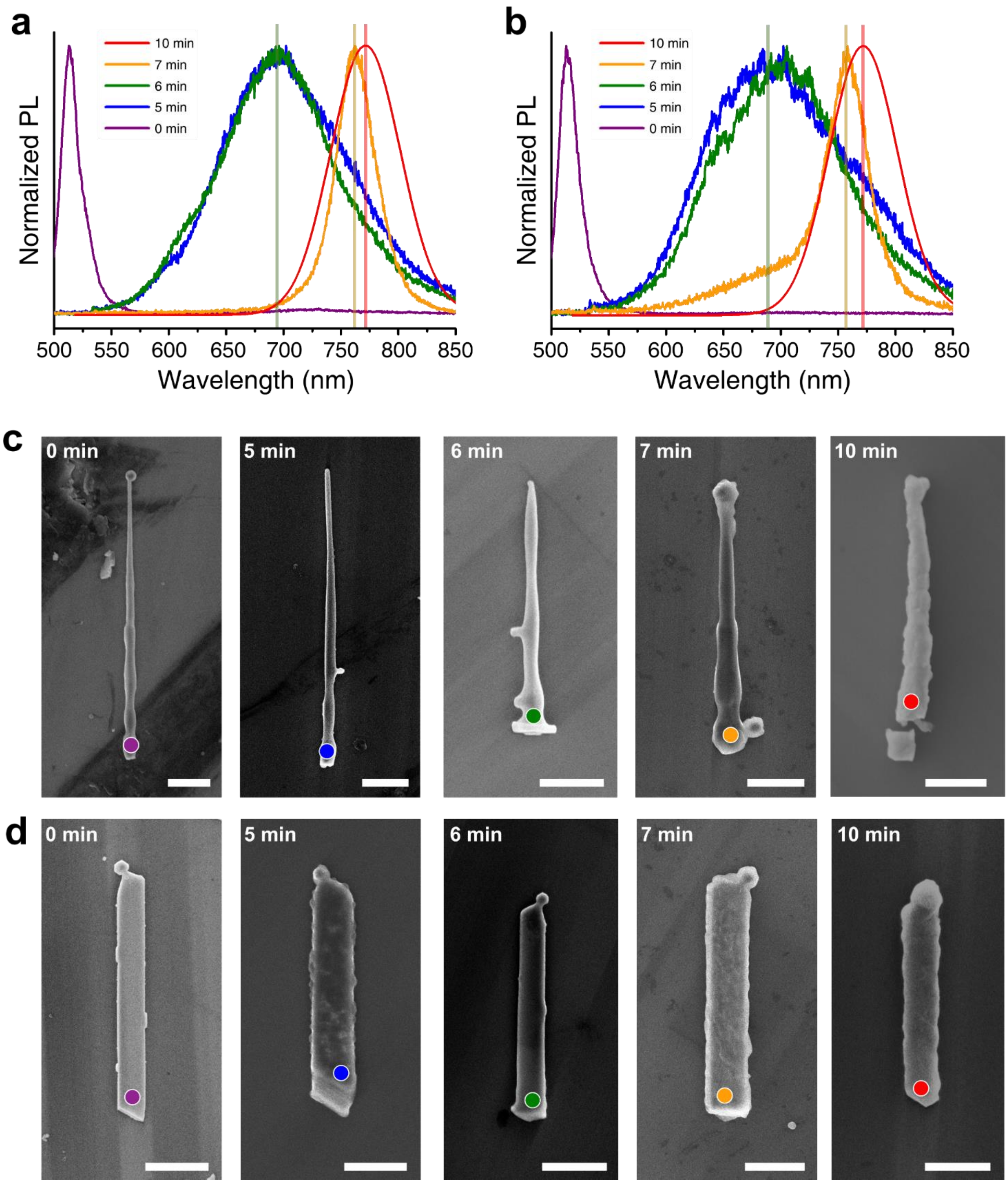

Figure S6. Normalized PL spectra measured at the bottom region of the nanowires of (a) [0001]-oriented type A and (b) [$\bar{1}2\bar{1}0$]-oriented type B, according to the various MAI conversion time of 0, 5, 6, 7, and 10 min. Characteristic wavelengths of 690, 760, and 772 nm were highlighted with green, yellow, and red vertical lines, respectively. (c, d) Representative SEM images of type A and B nanowires with various conversion times. Colored dots refer to the position where the PL spectra in (a) and (b) were obtained. Scale bars, 1 μm.

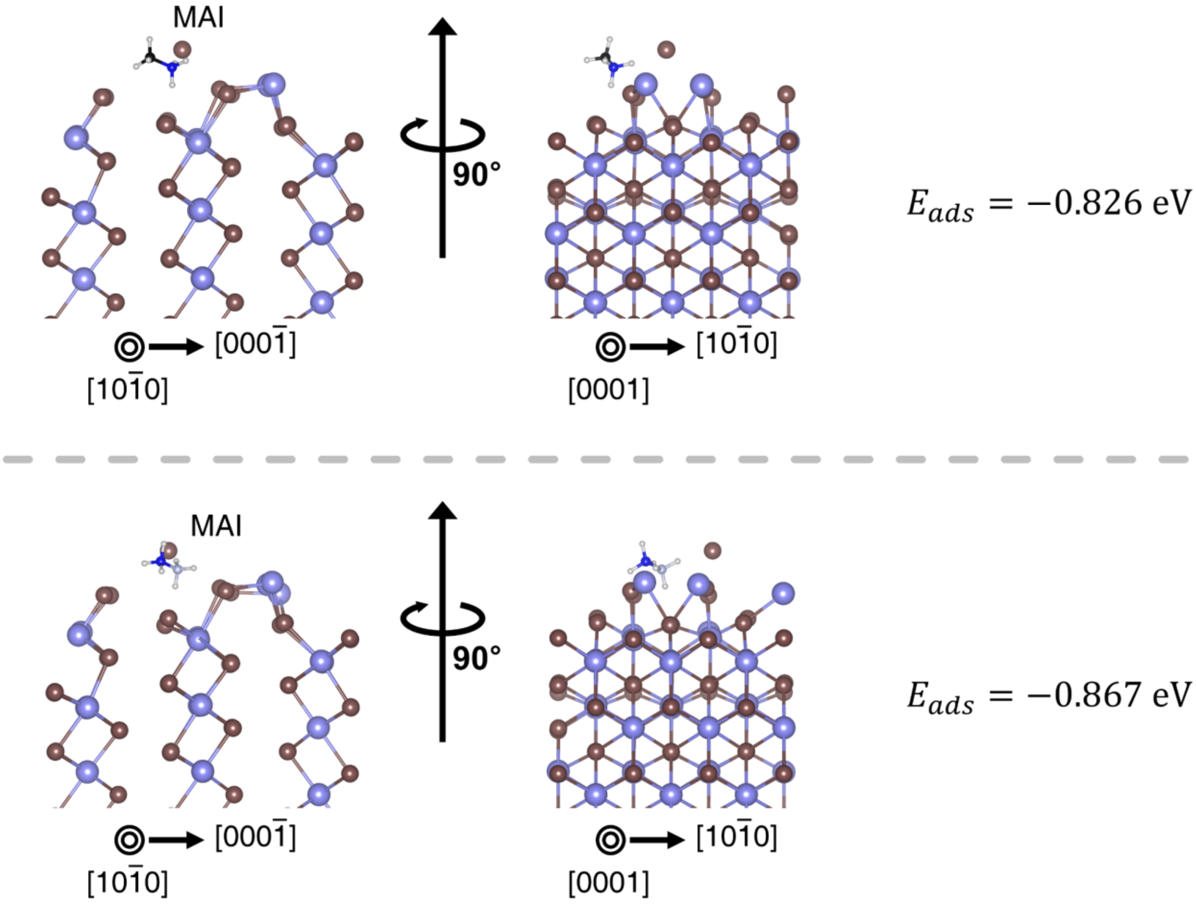

Figure S7. Different configurations for MAI adsorption on PbI_2 (01$\bar{1}$0) surfaces with corresponding adsorption energies.

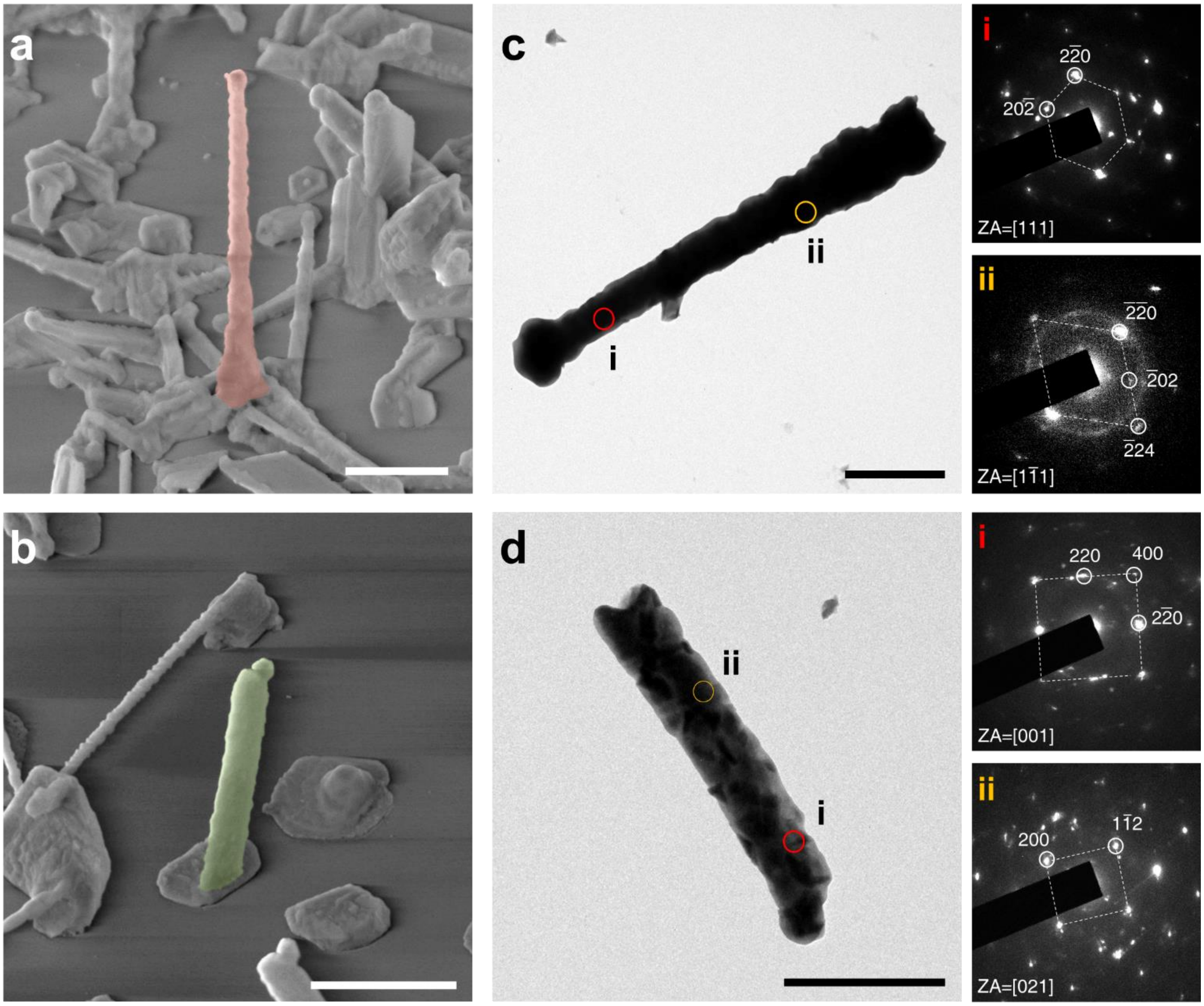

Figure S8. Representative tilt-view SEM images of $MAPbI_3$ nanowires converted from (a) [0001]-oriented type A and (b) $[\bar{1}2\bar{1}0]$-oriented type B nanowire. Scale bars, 2 μm. Low-magnification TEM images of $MAPbI_3$ nanowires converted from (c) type A and (d) type B nanowires, displayed with SAED patterns measured at two different locations in each sample.
